# A Rare Case of Total Talar Replacement for Talar Osteonecrosis Due to Fragility Fracture Secondary to Transient Osteoporosis of the Talus

**DOI:** 10.7759/cureus.78704

**Published:** 2025-02-07

**Authors:** Masatoshi Minami, Tetsuya Nakatani, Mahito Nishimura, Takafumi Onga, Noriyuki Kanzaki

**Affiliations:** 1 Department of Orthopaedic Surgery, Nakatani Orthopaedic Surgery Hospital, Kakogawa, JPN; 2 Department of Orthopaedic Surgery, Kobe University Graduate School of Medicine, Kobe, JPN

**Keywords:** bone atrophy, case report, medial malleolar fracture, talar fragility fracture, talar osteonecrosis, total talar replacement, transient osteoporosis

## Abstract

We report the case of a 44-year-old male patient without a notable medical history who developed transient osteoporosis following surgery for a medial malleolus fracture, which led to a stress fracture and osteonecrosis, ultimately requiring total talar replacement. The patient sustained the fracture spraining his ankle while welding. Osteosynthesis was performed using two cannulated cancellous screws, whereas bone union was achieved 3.5 months postsurgery. Five months after surgery, ankle joint pain worsened without new trauma episodes. Magnetic resonance imaging (MRI) showed diffuse hyperintensity over the entire talus on short tau inversion recovery (STIR) sequences, whereas radiography and computed tomography (CT) revealed fracture lines in the posterior talus. The fracture was initially treated with a non-load-bearing period; however, compression progressed, thereby leading to a diagnosis of talar osteonecrosis and total talar replacement. One year postsurgery, the Japanese Society for Surgery of the Foot ankle-hindfoot scale score improved from 24 presurgery to 85. The patient is currently being followed up. The Hounsfield units (HU) of the talus at the time of the fragility fracture, retrospectively measured via CT, were lower in areas with fragility fractures than in those with medial malleolar fractures, which suggests osteoporotic changes in the talus. Pathology of the excised talus confirmed osteoporosis with thinning of the trabecular bone, which was consistent with the MRI findings and suggested that transient osteoporosis led to fragility fractures and talar necrosis. The findings suggest that the delayed diagnosis of transient osteoporosis led to fragility fractures and osteonecrosis. This case highlights the importance of considering transient talar osteoporosis as a differential diagnosis in cases of persistent pain after ankle fracture surgery and performing an MRI early to guide treatment.

## Introduction

Transient osteoporosis primarily affects middle-aged men and, less commonly, women during the third trimester of pregnancy and the immediate postpartum period [1-3]. Transient osteoporosis usually presents with sudden-onset pain in weight-bearing areas, especially in the lower limb, often radiating distally. In most patients, transient osteoporosis leads to functional disability within four to eight weeks, followed by a gradual disappearance of the symptoms in the following six to 12 months. Clinical examination may reveal limited effusion. Magnetic resonance imaging (MRI) is fundamental for diagnosis, evidencing nonspecific and localized bone marrow edema hyperintense in T2 sequences. The principal treatment for transient osteoporosis is conservative management with limited weight-bearing, physical therapy, and nonsteroidal anti-inflammatory drugs (NSAIDs). Furthermore, treatment with bisphosphonates has shown promising results, shortening the duration of symptoms [1-3]. To date, only seven cases of transient osteoporosis in the talus have been reported in the literature [4-10]. This report describes the case of a patient who developed transient osteoporosis postsurgery for a medial malleolus fracture, which led to a stress fracture and osteonecrosis, ultimately requiring total talar replacement.

## Case presentation

**Figure 1:**
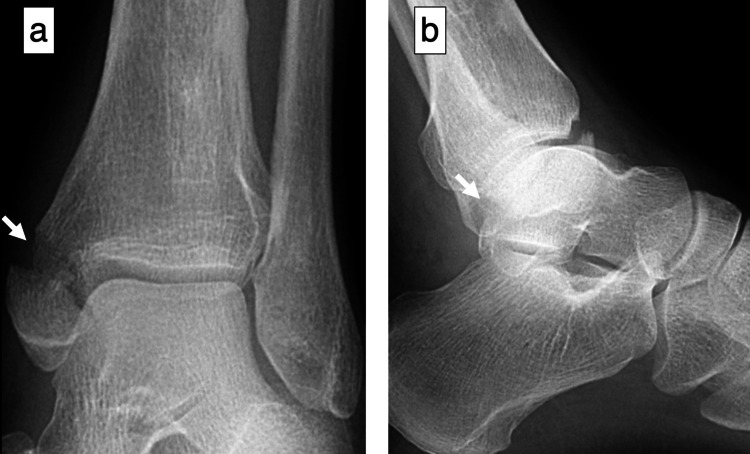
Preoperative radiographs of the left ankle (a) Anteroposterior view showing a medial malleolar fracture (arrow); (b) lateral view showing no talar fracture (arrow)

**Figure 2:**
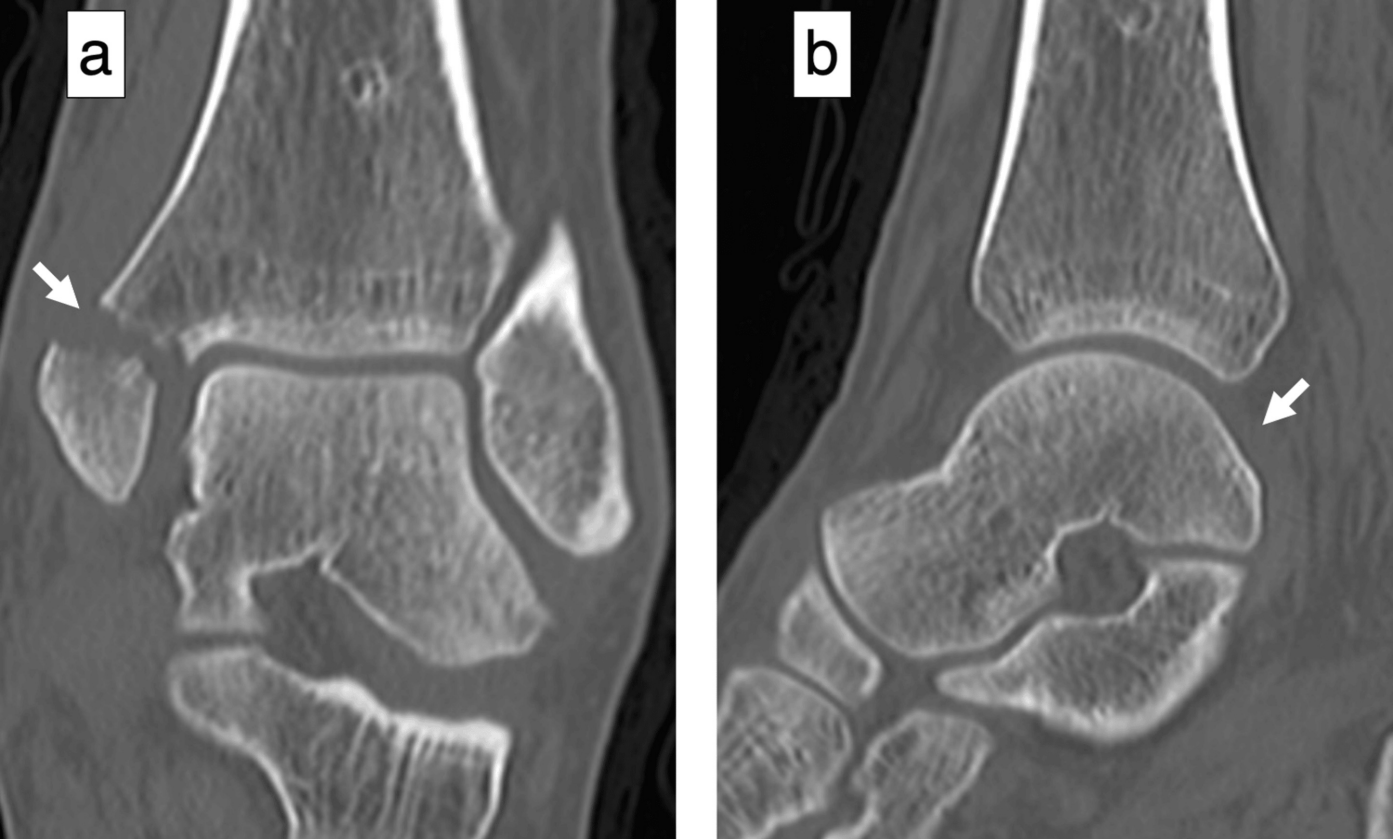
Preoperative computed tomography (a) Coronal section showing a medial malleolar fracture (arrow); (b) sagittal section showing no talar fracture (arrow)

**Figure 3:**
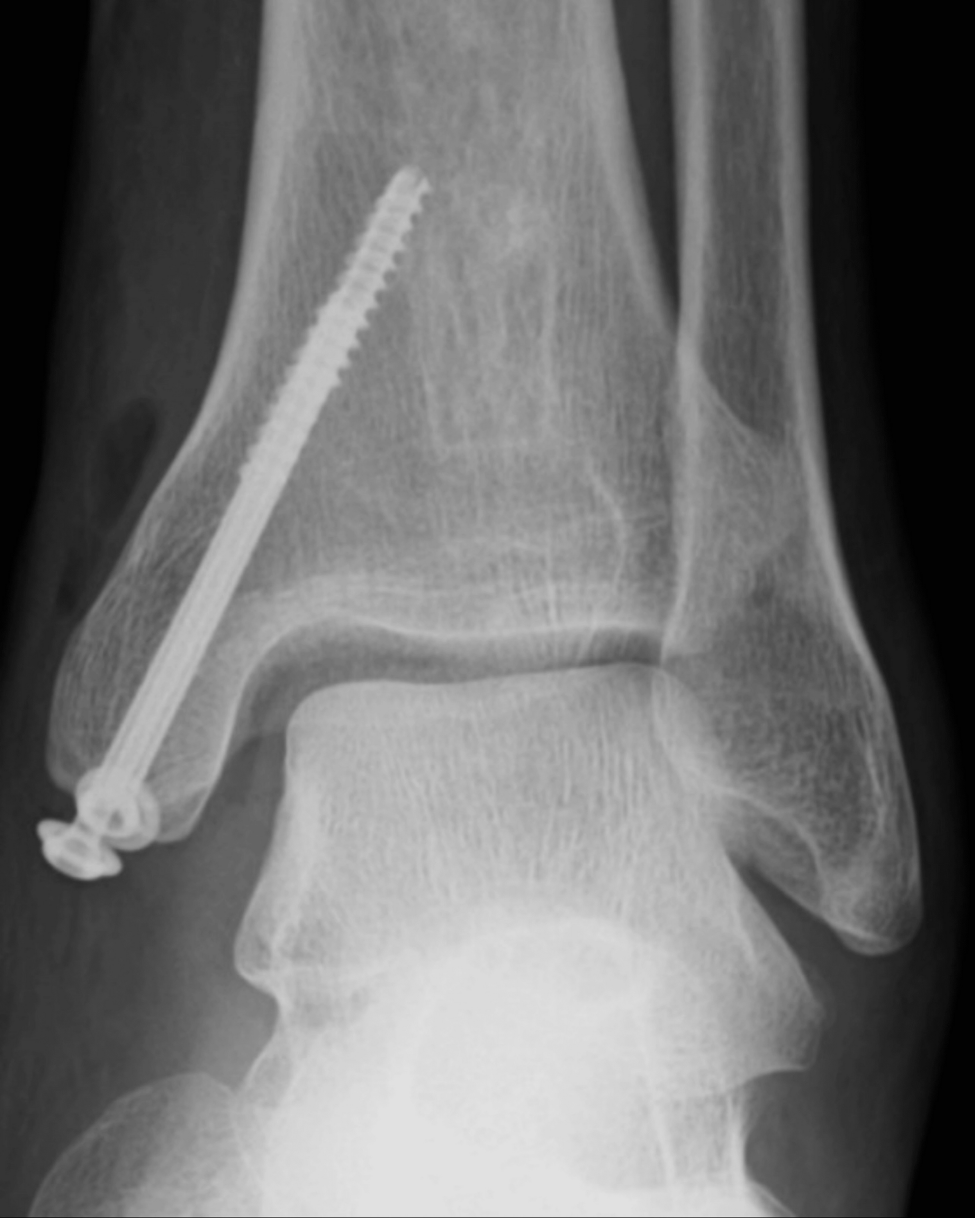
Postoperative radiograph of the left ankle Postoperative radiograph showing the anatomic fixation of a fracture with two cannulated cancellous screws

A 44-year-old male welder sprained his left ankle joint while jumping from a height of one meter while working. When he presented to a local doctor with left ankle pain, he was referred to our clinic for surgery owing to a medial malleolar fracture. The patient had no history of alcoholism, steroid use, smoking, hematological disease, or dyslipidemia. Radiographs and computed tomography (CT) scans at the initial visit to our hospital showed a medial malleolar fracture but no fibular or talar fractures (Figures 1, 2). The fracture was internally fixed using two cannulated cancellous screws (Figure 3).

**Figure 4:**
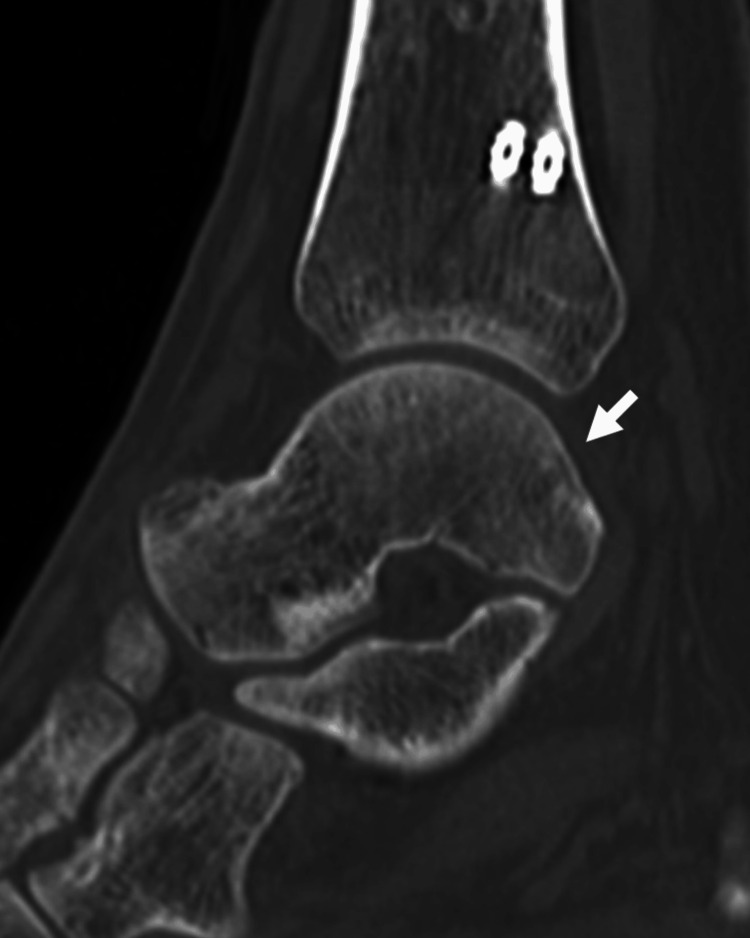
Computed tomography (sagittal section), 3.5 months postoperatively Computed tomography (sagittal section), 3.5 months postoperatively, showing no talar fracture (arrow)

Postoperative immobilization in a cast was performed for 10 days, followed by range-of-motion (ROM) and gait training with gradual weight-bearing. No signs of allodynia, hyperalgesia, abnormal sweating, and changes in skin color were observed, and rehabilitation, including ROM training, continued. The left ankle joint pain under load improved but persisted 3.5 months postsurgery. A CT revealed bone union at the medial malleolar fracture site, with no fibular or talar fractures that could explain the pain during weight-bearing (Figure 4).

**Figure 5:**
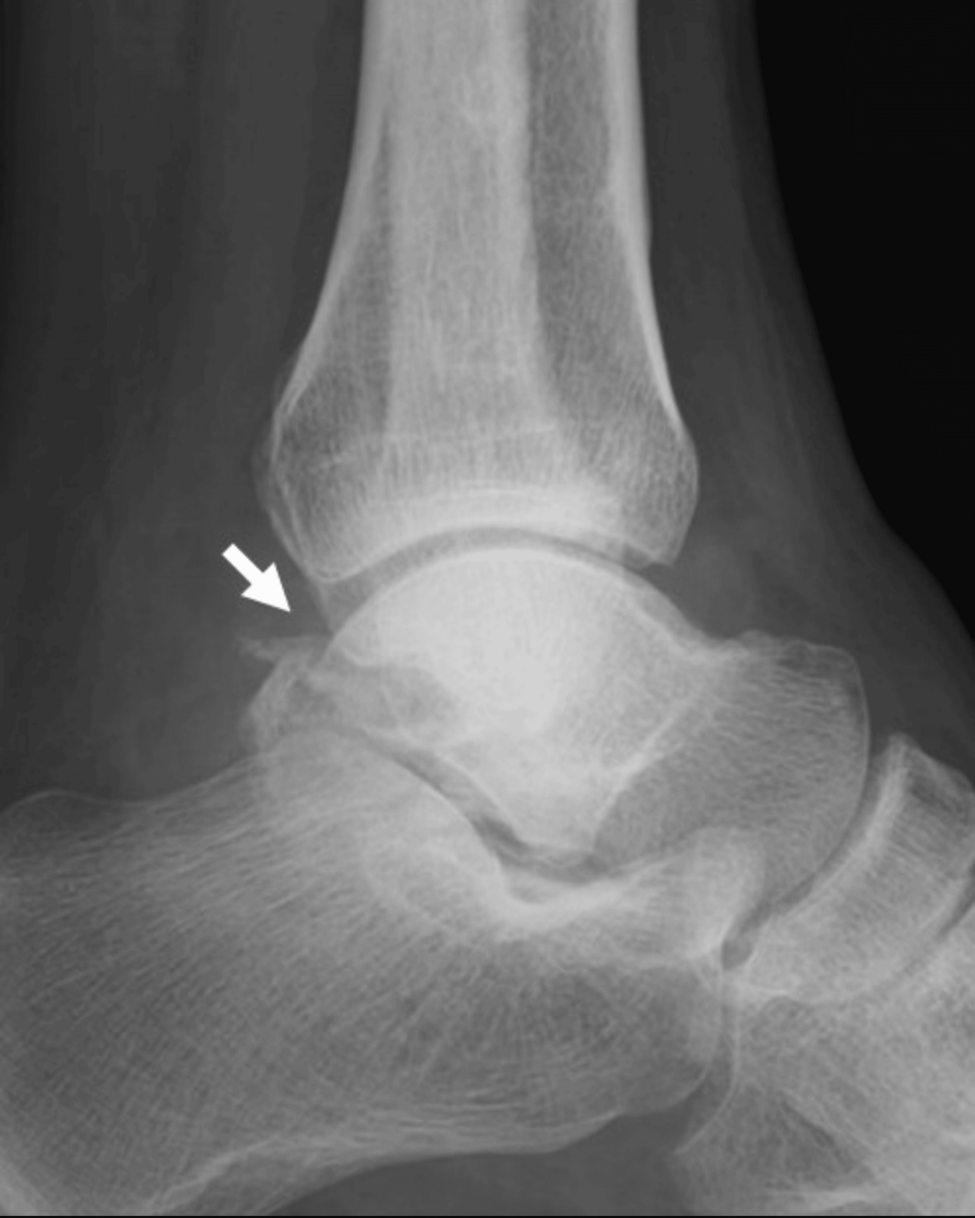
Radiograph, six months postoperatively Radiograph, six months postoperatively, showing irregularity of the posterior part of the talar dome (arrow)

Five months postoperatively, approximately two weeks after returning to work as a welder, the patient experienced worsened left ankle joint pain, despite no new episodes of trauma. Six months postoperatively, radiography showed irregularity of the posterior part of the dome of the talus (Figure 5).

**Figure 6:**
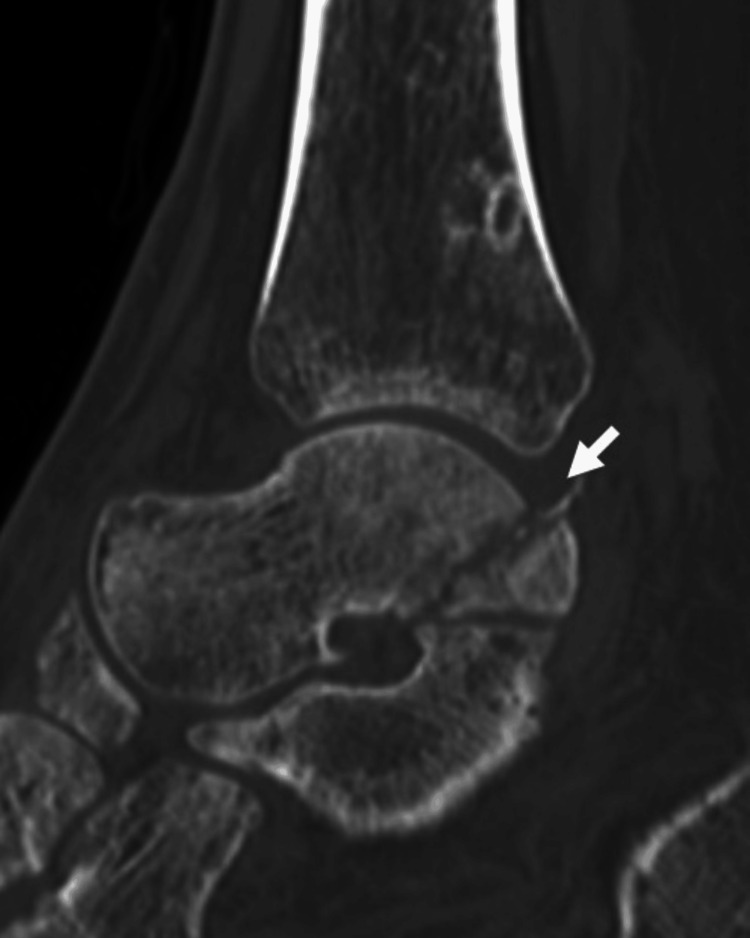
Computed tomography (sagittal section), six months postoperatively Computed tomography (sagittal section), six months postoperatively, showing bone atrophy of the talus and a fracture without osteosclerosis at the posterior tuberosity of the talus (arrow)

**Figure 7:**
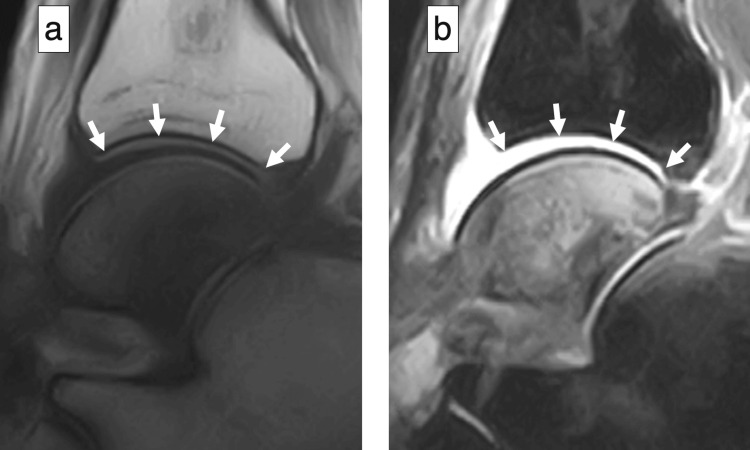
MRI (sagittal sections), six months postoperatively STIR: short tau inversion recovery (a) T1-weighted imaging showing diffuse low-signal intensity (arrows); (b) T2-weighted STIR imaging showing diffuse hyperintense signal within the talus with effusion (arrows)

CT showed bone atrophy of the talus and a fracture at the posterior tuberosity of the talus without osteosclerosis (Figure 6), whereas MRI revealed diffuse low-signal T1 and high-signal short tau inversion recovery (STIR) intensity changes throughout the talus (Figure 7).

**Figure 8:**
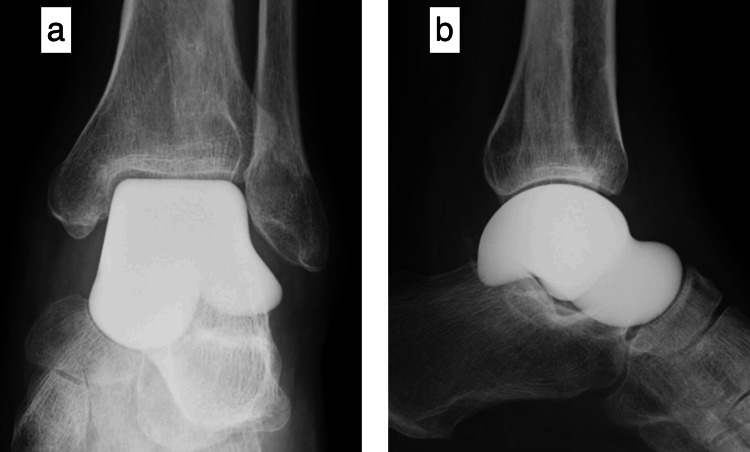
Postoperative radiographs of total talar replacement (a) Anteroposterior view; (b) lateral view

**Figure 9:**
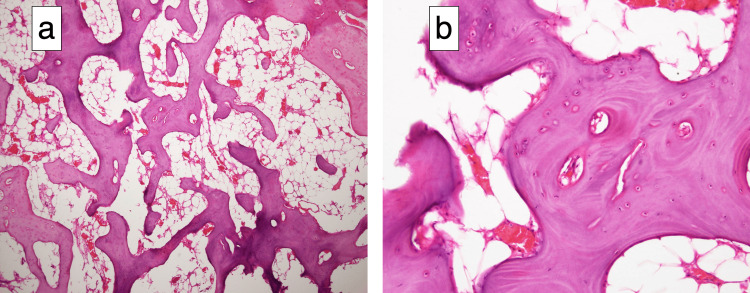
Histological findings of the excised talus Histological findings of the excised talus at (a) 4× and (b) 20× magnification (hematoxylin and eosin staining). The substrate was basophilically stained in the slightly narrower portion of the trabecular bone, the staining of the osteocyte nuclei was lost, and no osteoblasts or osteoclasts were seen

At the time of the initial injury, no talar fracture was evident on radiography or CT. Similarly, 3.5 months after the injury, radiography and CT showed no signs of talar fracture, despite the patient's weight-bearing gait. Therefore, we concluded that a fragility fracture likely occurred less than six months after the initial injury, which led to secondary osteonecrosis of the talus. Given the patient's severe pain and desire to preserve significant joint mobility, total talar replacement was performed instead of arthrodesis (Figure 8). The pathology of the excised talus revealed that the substrate was basophilically stained in a slightly narrower portion of the trabecular bone, the osteocyte nuclei showed loss of staining, whereas no osteoblasts or osteoclasts were observed (Figure 9). Four months after total talar replacement, the patient returned to work as a welder. At 1.5 years postsurgery, the Japanese Society for Surgery of the Foot Ankle and Hindfoot Scale scores [11,12] improved from 24 preoperatively to 85. The patient is currently being followed up.

## Discussion

Osteonecrosis is a condition in which the bone tissue becomes devitalized and can progress to degradation and structural collapse. It predominantly results from traumatic injuries, with the hip being the most common site, particularly after femoral neck fractures and hip dislocations. Traumatic osteonecrosis is also commonly associated with talar fractures in the feet and ankles. In addition to posttraumatic causes, nontraumatic factors such as alcoholism, steroid use, smoking, hematologic disorders, dyslipidemia, and idiopathic conditions can occasionally contribute to its development [13]. Considering the pathophysiology of this case, it is reasonable to assume that no subclinical fracture of the talus occurred at the time of the initial injury, as no evidence of a talar fracture was observed on radiography or CT at the time of the medial malleolar fracture. Additionally, no indication of a fracture line or osteosclerosis existed, thus indicating healing on radiographs or CT scans 3.5 months after the injury. In this case, the medial malleolar fracture was simple, with no evidence of dislocation or tibiofibular ligament injury, and was not caused by high-energy trauma. Therefore, it is unlikely that the talus’s vascular supply was compromised and led to osteonecrosis. The patient had no history of alcoholism, steroid use, smoking, hematological disease, or dyslipidemia. This made the risk of osteonecrosis from traumatic, vascular, intrinsic dietary, or environmental factors extremely low.

This report used the method described by Lee et al. to measure the Hounsfield units (HU) of the talus using CT [14]. The results showed that the HU was 414.40 at the time of injury, decreased to 407.11 at 3.5 months, and further decreased to 353.15 six months after the injury, thus indicating ongoing bone atrophy. Histopathological examination of the excised talus revealed basophilic staining of the narrow trabecular bone, loss of osteocyte nuclei staining, and absence of osteoblasts or osteoclasts. These pathological findings suggest the presence of bone atrophy. The results of this case were likely to indicate an extremely rare fragility fracture of the talar bone, which may have resulted in osteonecrosis. Fragility fractures of the talus are rare, and only one previous English-language study addressed this topic, without discussing the causes of such fractures [15].

When considering the cause of early postoperative bone atrophy in this patient, we first considered complex regional pain syndrome (CRPS), as bone atrophy developed following trauma to the ankle joint fracture. This is because in the acute phase of CRPS, inflammatory cytokines such as tumor necrosis factor (TNF)-α, interleukin (IL)1B, and IL-17 activate osteoblasts and osteoclasts, causing rapid bone turnover [16]. However, although the patient experienced postoperative ankle joint pain, he did not exhibit abnormal skin color, sweating, or altered sensation and was able to undergo rehabilitation, including ROM training, similar to other postoperative patients. Therefore, he did not meet the diagnostic criteria for CRPS [16]. Second, the possibility of bone atrophy caused by unloading was considered. The patient underwent three weeks of cast immobilization postoperatively, during which he was unloaded. Partial loading was initiated four weeks postoperatively. Although the increase in loading was slower than usual because of pain, disuse osteoporosis is unlikely to explain fragility fracture, as significant osteoporosis would not typically develop after only one month of unloading.

Transient osteoporosis is generally considered idiopathic and not triggered by trauma. However, El Masry et al. described a case of transient osteoporosis of the knee that developed as a result of trauma [17]. In this case, transient osteoporosis developed in the talus after ankle fracture surgery. The fragility fracture of the talus occurred as the result of a delayed transient osteoporosis diagnosis and permission to return to work without an exemption order, despite persistent pain following ankle fracture surgery. Despite the fragility of the talus, osteonecrosis was attributed to a fragility fracture due to the patient’s return to work. A welder by profession, the patient desired to preserve joint mobility; therefore, this led to the decision to select total talus replacement instead of arthrodesis. The postoperative course is currently favorable 1.5 years postsurgery.

## Conclusions

We report a rare case of osteonecrosis due to a fragility fracture following a medial malleolar fracture of the ankle joint, which required total talar replacement. The fragility fracture was likely caused by a delay in diagnosis. In cases of persistent postoperative pain after an ankle fracture, early CT and MRI should be performed to assess the possibility of transient osteoporosis or talar fragility fractures.
